# Gut microbial and metabolomic alterations associated with herpetic trigeminal neuralgia: an exploratory multi-omics study

**DOI:** 10.22514/jofph.2026.067

**Published:** 2026-09-12

**Authors:** Mingyue Tian, Yangyang Wang, Cong Li, Jiaming Fan, Qing Chen, Huilian Bu, Xiaochong Fan, Qingying Liu

**Affiliations:** ^1^Department of Pain Medicine, The First Affiliated Hospital of Zhengzhou University, 450052 Zhengzhou, Henan, China

**Keywords:** Herpetic trigeminal neuralgia, Gut microbiota, Metagenomics, Metabolomics, Gut–brain axis

## Abstract

**Background**: Herpetic trigeminal neuralgia (HTN) is a neuropathic pain disorder caused by varicella-zoster virus invasion of the trigeminal nerve, but its mechanisms remain poorly elucidated. Accumulating evidence implicates gut microbiota dysbiosis and metabolic perturbations in modulating chronic pain via the gut–brain axis, yet their roles in HTN have not been systematically explored. **Methods**: Integrated multi-omics analysis was performed on 19 patients with HTN and 18 healthy controls. Fecal metagenomic sequencing characterized gut microbial composition and function. Untargeted metabolomics profiled metabolic signatures in fecal and circulating samples. Weighted gene co-expression network analysis (WGCNA) and random forest modeling screened pivotal microbial and metabolic features associated with HTN. Correlation analysis between differential metabolites and clinical pain scores validated the translational potential of identified alterations. **Results**: HTN patients showed significantly reduced gut microbial diversity, with enriched *Proteobacteria*, depleted butyrate-producing bacteria (including *Faecalibacterium*, *Roseburia*, and *Lachnospira*), and expanded opportunistic pathogens (*Escherichia coli* and [*Ruminococcus*] *gnavus*). Microbial pathways for pH homeostasis and oxidative stress defense were downregulated. Metabolomic analysis identified differential metabolites, primarily involving nicotinate/nicotinamide metabolism and retrograde endocannabinoid signaling. WGCNA and random forest analyses suggested fecal metabolites as potential candidate features, with associations between gut microbial dysregulation and systemic metabolic changes. Furthermore, screened metabolites correlated significantly with pain scores. **Conclusions**: This study provides multi-omics evidence of gut microbial and metabolic dysregulation in HTN, generating hypotheses for further mechanistic research. Large-scale, multicenter prospective cohorts with standardized metadata collection are needed to validate these findings.

## 1. Introduction

Herpetic trigeminal neuralgia (HTN) is a debilitating neuropathic pain disorder arising from varicella-zoster virus (VZV) reactivation in the trigeminal nerve. Following primary infection, the virus establishes lifelong latency in the trigeminal ganglia. With waning VZV-specific immunity, the virus may reactivate to produce herpes zoster (HZ)—a painful dermatomal rash—and the trigeminal nerve is involved in the majority (58%) of craniofacial HZ cases.

According to the third edition of the International Classification of Headache Disorders (ICHD-3), HTN is defined as pain lasting ≥1 month in the trigeminal territory following herpes zoster infection. Characteristic pain features include burning sensations and pruritus, and patients often exhibit marked sensory loss and brush-evoked mechanical allodynia within the affected trigeminal territory. In some patients, sensory deficits are less apparent, whereas hypersensitivity to thermal or pinprick stimulation may predominate.

In severe cases, the disease can profoundly impair eating, speaking, and daily activities, imposing a considerable psychological and physiological burden on patients. Global epidemiological data indicate that the incidence of postherpetic neuralgia is approximately 18% among individuals aged over 50 years, whereas the corresponding prevalence reaches 33% in those aged >80 years [1]. Trigeminal herpes zoster accounts for approximately 15%–20% of all herpes zoster cases. Given its complex pathophysiological mechanisms and relatively high incidence, HTN has emerged as a major public health concern that demands urgent attention [2].

HTN remains challenging to manage, and currently no curative therapy is available. Vaccination is commonly recommended for high-risk populations for prevention. Patients receiving treatment within nine months of disease onset may achieve better prognostic outcomes, whereas pain refractory to conservative therapies often necessitates interventional procedures [3].

In recent years, the role of the gut microbiota in neurological diseases has attracted extensive attention. The gut microbiota is indispensable for maintaining multiple physiological functions of the human body, particularly the regulation of the central nervous system (CNS). Its modulatory effects on brain physiology are primarily manifested in the regulation of synaptogenesis, neurotransmitter synthesis, and the expression of neurotrophic factors, including brain-derived neurotrophic factor and nerve growth factor-A1. In addition, blood–brain barrier (BBB) integrity is a prerequisite for normal CNS development, as it constructs a stable and optimal microenvironment for the growth and differentiation of neurons [4].

Mounting evidence indicates that gut microbiota dysregulation drives disease progression across different stages of neuroinflammation and neurodegeneration, directly facilitating the onset and progression of multiple neurological disorders [5, 6]. In contrast, specific bacterial taxa, especially probiotic-related gut microorganisms, have been validated to exert therapeutic effects on numerous neurological diseases [7]. Existing studies further support that gut microbial dysbiosis plays a critical role in the initiation and progression of human diseases, including neurodegenerative disorders. Accordingly, targeting gut microbiota to mitigate disease risk, improve clinical prognosis, and characterize microbial ecological signatures has emerged as a prominent and rapidly advancing research direction in translational medicine [5].

Emerging clinical evidence has demonstrated significant gut microbiota changes in patients with postherpetic neuralgia [8]. Nevertheless, gut microbiota-related research specifically focusing on HTN remains scarce. Therefore, the present study recruited clinically diagnosed HTN patients and profiled their gut microbiota and corresponding metabolites, aiming to elucidate the potential pathogenic mechanisms underlying HTN. Furthermore, this study attempted to screen candidate microbial and metabolic markers, which may offer valuable insights for future mechanistic exploration and clinical management of HTN.

## 2. Materials and methods

### 2.1 Participant recruitment

This study recruited healthy controls (HC) without known diseases and patients diagnosed with HTN from the First Affiliated Hospital of Zhengzhou University (Henan, China) between October 2025 and January 2026. The clinical diagnosis of HTN was strictly formulated in accordance with the ICHD-3 diagnosis criteria. Fecal and blood samples were collected from all enrolled participants, and baseline demographic and clinical information, including age, sex, and body mass index (BMI), was systematically recorded. For HTN patients, clinical pain severity was evaluated via the Visual Analogue Scale (VAS), with scores documented on the day of sample collection. Additionally, disease duration and the distribution of affected trigeminal nerve branches were comprehensively recorded. A total of 37 participants were enrolled in this study, consisting of 18 HC and 19 HTN patients.

### 2.2 Inclusion and exclusion criteria

The inclusion criteria were as follows for HTN patients: (1) Consistent with the diagnostic guidelines of the ICHD-3 [9], enrolled patients were diagnosed with two subtypes of herpes zoster-related trigeminal neuralgia: (a) Painful trigeminal neuropathy attributed to herpes zoster (ICHD-3 13.1.2.1, Unilateral facial pain of less than three months’ duration but more than 1 month’s in the distribution(s) of one or more branches of the trigeminal nerve, caused by and associated with other symptoms and/or clinical signs of acute herpes zoster); (b) Trigeminal postherpetic neuralgia (ICHD-3 13.1.2.1): Unilateral facial pain persisting or recurring for at least three months in the distribution(s) of one or more branches of the trigeminal nerve, with variable sensory changes, caused by herpes zoster; (2) Aged between 34 and 77 years; (3) Moderate to severe pain with a VAS score ≥4; and (4) Disease duration ≥1 month.

The inclusion criteria for HC were: (1) Age-matched with HTN patients (34–77 years old) with consistent gender distribution; (2) No personal history of herpes zoster, trigeminal neuralgia, or any other neuropathic pain disorder; and (3) Free from severe systemic diseases, including diabetes mellitus, autoimmune disorders, malignant tumors, and active infectious diseases.

The exclusion criteria applicable to all participants were as follows: (1) A confirmed diagnosis of irritable bowel syndrome (IBS) or inflammatory bowel disease (IBD); (2) Clinical presentation with diarrhea symptoms; (3) A definitive diagnosis of any psychiatric disorders; and (4) A history of active smoking or alcohol dependence.

### 2.3 Sample collection and sequencing

Fecal samples were immediately frozen at −80 ℃ after collection and processed for metagenomic and metabolomic sequencing within one month. Whole blood samples were processed promptly to isolate serum, which was immediately aliquoted and preserved at −80 ℃ for subsequent metabolomic analysis. All metabolomic sequencing procedures were completed within one month following sample preservation to ensure sample stability and data reliability.

### 2.4 Metagenomic sequencing and untargeted metabolomics (LC–MS/MS) analysis

Metagenomic sequencing was performed by Shanghai Biotree Biomedical Technology Co., Ltd. All sequencing procedures were implemented on the Illumina NovaSeq 6000 platform (San Diego, CA, USA). Briefly, total microbial DNA was extracted from qualified fecal samples and subjected to rigorous quality control. Raw sequencing data were generated in FASTA with Quality scores (FASTQ) format, and the acquired reads were subsequently corrected and assembled into contigs. After dereplication, gene prediction was conducted to construct a nonredundant amino acid sequence dataset. Multiple public databases were adopted for the functional annotation of amino acid sequences, yielding functional abundance profiles at distinct hierarchical levels, which supported the subsequent analyses of microbial functional composition, diversity, and differential enrichment. Furthermore, high-quality datasets and nonredundant amino acid sequences were used for taxonomic annotation, thereby characterizing microbial community profiles at the species and subspecies levels.

Untargeted metabolomic analysis based on Liquid Chromatography-Tandem Mass Spectrometry (LC–MS/MS) was also performed by Shanghai Biotree Biomedical Technology Co., Ltd. Metabolite separation was implemented using an ultra-high-performance liquid chromatography system (Vanquish, Thermo Fisher Scientific, Waltham, MA, USA) equipped with a Waters ACQUITY Premier HSS T3 column (1.8 μm, 2.1 × 100 mm). The mobile phases consisted of aqueous phase A (ultrapure water with 0.1% formic acid) and organic phase B (acetonitrile containing 0.1% formic acid). For data preprocessing, metabolite features with missing values exceeding 50% were excluded from downstream analyses. Remaining features with a missing value proportion of ≤50% were imputed using the k-nearest neighbor (KNN) algorithm (k = 10), where missing values were estimated based on the weighted average of measurements from adjacent samples within the same group [10].

Metabolite annotation was accomplished by matching acquired MS/MS spectra against the proprietary database of Biotree Biotech Co., Ltd (Shanghai, China), with supplementary verification from public databases, including the Human Metabolome Database (HMDB) and Kyoto Encyclopedia of Genes and Genomes (KEGG). Metabolite identification confidence was categorized following the standardized criteria proposed by the Metabolomics Standards Initiative (MSI). Specifically, level 1 (Confident Identification) required consistent matching of both retention time and MS/MS fragment profiles with authentic reference standards. Level 2 (Putative Identification) was defined by spectral matching against public databases (*e.g.*, HMDB, METLIN (A metabolite mass spectral database)). Level 3 (Tentative Annotation) relied solely on accurate mass matching. All metabolites identified in this study satisfied Level 2 identification standard [11].

### 2.5 Data analysis

For microbiome data analysis, alpha and beta diversity analyses were conducted using the vegan and picante packages in R. Phylogenetic trees were constructed and visualized with the ggtree, ape, ggstar, and ggplot2 packages. Linear discriminant analysis effect size (LEfSe) was performed using the microeco R package to identify differential microbial features between groups. Differential metabolite analysis was implemented using the limma R package. Linear models were fitted with lmFit, and intergroup contrast matrices were constructed. Heatmaps of differential metabolites were generated using ggplot2. Volcano plots were constructed with log_2_ fold change (log_2_FC) on the x-axis and −log_10_ (*p*-value) on the y-axis, while *p*-values were corrected using the Benjamini-Hochberg (BH) method with false discovery rate (FDR) <0.05. For metagenomic sequencing data, the LEfSe algorithm was applied, and the Kruskal-Wallis test was used to identify differentially abundant bacterial taxa between groups (*p* < 0.05). Gene Ontology (GO) functional annotation of metagenomic sequences was performed using Blast2GO [12], with the non-redundant (NR) protein database as the reference. Genes were annotated into three core GO categories, including biological process (BP), molecular function (MF), and cellular component (CC), to establish gene-function correspondence [13].

The GO annotation results were then integrated with the transcripts per million (TPM) abundance matrix. For each GO term, TPM values of all assigned genes were summed to construct a GO term-level abundance matrix, thereby minimizing the influence of sequencing depth differences on intergroup comparisons. Metabolic KEGG enrichment analysis of metabolites was conducted using the clusterProfiler R package (version 4.16.0), with functional annotation reference to the KEGG database. The Wilcoxon rank-sum test was used to evaluate the differential alterations between the HC and HTN groups. Multiple testing correction was performed using the BH method. GO terms and KEGG pathways with adjusted *p* < 0.05 were defined as statistically significantly enriched.

Given the exploratory design and the limited sample size of this study, all machine-learning analyses, including least absolute shrinkage and selection operator (LASSO), random forest, and WGCNA, were performed for hypothesis generation rather than confirmatory validation. WGCNA was specifically applied to explore potential modular correlations among differential serum and fecal metabolites, and the sample size adopted in this study complied with the empirical reference range recommended by the official guideline [14]. Differential serum metabolites and the top 150 fecal metabolites ranked by variance were incorporated for network construction. The optimal soft-thresholding power was determined as 11 based on the scale-free topology criterion (Scale-Free Topology Model Fit *R*^2^ ≥ 0.8), and a signed weighted co-expression network was subsequently established. Module partitioning was conducted using the blockwiseModules function, with the minimum module size set to 10 and the module merging threshold (mergeCutHeight) set to 0.25.

For LASSO regression analysis, all 37 enrolled participants were included to construct a binary diagnostic model for distinguishing HTN patients from healthy controls. The full dataset was randomly partitioned into a training set (n = 26) for model construction and an internal test set (n = 11) for preliminary validation.

Module eigengenes (MEs) were subsequently calculated and correlated with clinical phenotype, with HTN status encoded as a binary variable (0/1), using Pearson correlation analysis. Phenotype-related key modules were screened according to module–trait correlation coefficients (*r*) and corresponding *p*-values. For each key module, module membership (MM; representing the correlation of individual metabolites with MEs) and gene significance (GS; representing metabolites-phenotype correlation) were quantified. GS–MM scatter plots were generated to verify the biological validity of core modules. Furthermore, metabolites within individual modules were ranked by absolute GS values to screen core metabolites strongly correlated with the HTN phenotypes.

Random forest regression models were constructed for exploratory analysis to characterize potential associations between differential microorganisms and metabolites. The relative abundances of the 47 differential microbial taxa were defined as independent variables, while metabolite abundances were defined as dependent variables. Separate random forest models were built for each metabolite using 500 trees (ntree = 500) with a random seed set to 1234.

Model performance was evaluated using the coefficient of determination (*R*^2^), root mean square error (RMSE), and mean absolute error (MAE). Leave-one-out cross-validation (LOOCV) was performed to compute cross-validated mean squared error (CV-MSE), and the relative error was calculated as the ratio of CV-RMSE to the observed mean value for evaluating model generalizability. Metabolites models with *R*^2^ > 0.5 and relative error <0.5 were regarded as statistically reliable. Feature importance scores (%IncMSE) were extracted to quantify the explanatory power of individual microbial taxa. The top 20 metabolites with the highest cumulative %IncMSE scores were selected for visualization, and stacked bar plots were generated to delineate the relative contributions of different microbial taxa to metabolite variation.

Spearman’s rank correlation analysis was conducted to explore the relationships between the relative abundances of microbial genera and VAS pain scores. Pain scores were defined as independent variables, and microbial relative abundances were set as dependent variables. Spearman correlation coefficients (*ρ*) and corresponding *p*-values were calculated for each microbial genus, with the BH method applied for multiple testing correction. Scatter plots with fitted linear regression trend lines and 95% confidence intervals (CI) were generated for visualization.

All R-based analyses and visualizations were implemented in RStudio (version 2025.05.1).

Baseline clinical data were analyzed using SPSS 30.0 (IBM, Armonk, NY, USA). Specifically, age and BMI differences between the two groups were compared using an independent-samples *t*-test, while gender distribution was assessed using the chi-square (χ^2^) test.

## 3. Results

### 3.1 HTN is associated with the altered gut microbiota composition

Baseline demographic and clinical characteristics of all enrolled participants are presented in Table 1. No significant intergroup differences in age, gender, or BMI were observed between the HTN and HC groups (all *p* > 0.05), indicating well-matched baseline conditions for subsequent comparative analysis.

**Table 1.** Baseline clinical characteristics of the study participants.

Characteristic	HTN (n = 19)	HC (n = 18)	*p*-value
Age (yr), mean ± SD	55.1 ± 14.0	54.9 ± 14.4	0.98
Sex (male), n (%)	11 (57.9)	10 (55.6)	0.89
BMI (kg/m^2^), mean ± SD	23.5 ± 1.9	23.6 ± 1.7	0.97
Disease duration (d), mean ± SD	55.4 ± 30.8	-	-
VAS score, mean ± SD	5.3 ± 1.0	-	-
Affected branch V1, n (%)	11 (57.9)	-	-
Affected branch V2, n (%)	5 (26.3)	-	-
Affected branch V1 + V2, n (%)	2 (10.5)	-	-
Affected branch V1 + V2 + V3, n (%)	1 (5.3)	-	-

*HTN: Herpetic trigeminal neuralgia; HC: healthy controls; BMI: body mass index; VAS: Visual Analogue Scale; SD: Standard Deviation.*

Fecal metagenomic sequencing was performed to profile gut microbial compositional and functional alterations in HTN patients. Significant reductions in microbial αdiversity were detected in the HTN group, as reflected by decreased Gini-Simpson index and Shannon index (*p* < 0.05; Fig. 1a).

The Shannon index comprehensively reflects microbial community richness and evenness, therefore, its reduction indicated simultaneous declines in species richness and uniform distribution in the gut microbiota of HTN patients. The Gini-Simpson index primarily characterizes the diversity of dominant taxa; its decrease suggested simplified community structure and reduced diversity of dominant microbial populations in HTN patients. Although the Observed Species index did not reach statistical significance, a consistent declining trend in species richness was observed in the HTN group.

Principal coordinate analysis (PCoA) based on metagenomic data was further conducted to evaluate intergroup differences in β-diversity (Fig. 1b). The overall gut microbial community structure differed significantly between HTN patients and HC (*p* = 0.01), implying that HTN development is closely associated with global alterations in gut microbiota composition.

Partial overlap of confidence ellipses between the two groups in the two-dimensional PCoA plot, nevertheless, a clear intergroup separation trend was identified. HC samples exhibited tight clustering and were predominantly distributed on the left axis, indicating stable gut microbial composition with lower individual variability. In contrast, HTN samples were broadly dispersed across multiple quadrants, demonstrating increased interindividual heterogeneity and disrupted gut microbiota homeostasis.

LEfSe analysis was further applied to screen characteristic gut microbial taxa that distinguished the two groups. The cladogram results revealed that differential microbial taxa were predominantly enriched in the HC group (Fig. 1c). Using an linear discriminant analysis (LDA) score >3 and an adjusted *p*-value (padj) < 0.05 as screening criteria, a total of 89 characteristic microbial taxa were identified, and the top 30 ranked by LDA score were visualized (Fig. 1d).

**Fig. 1.** Gut microbial diversity and differential taxa between healthy controls (HC) and patients with herpetic trigeminal neuralgia (HTN). (a) Boxplot of microbial alpha diversity. *indicates significant differences between groups. (b) Microbial beta diversity between HC and HTN groups. (c) Phylogenetic tree of LEfSe differential analysis. (d) LDA score plot of LEfSe differential analysis. *indicates a statistically significant difference (*, *p* < 0.05); **indicates a highly statistically significant difference (**, *p* < 0.01). PCoA: Principal coordinate analysis; PERMANOVA: Permutational Multivariate Analysis of Variance; LDA: Linear Discriminant Analysis.

At the phylum level, *Proteobacteria* and *Firmicutes* were identified as the primary differential taxa between the two groups. *Proteobacteria* were markedly enriched in the HTN group, whereas Firmicutes predominated in HC group.

At the genus level, the HC group showed significant enrichment of *Faecalibacterium*, *Blautia*, *Roseburia*, *Dialister*, and *Anaerobutyricum*. In comparison, the HTN group exhibited increased abundance of *Mediterraneibacter* and *Escherichia*, which contain multiple opportunistic pathogenic species.

At the species level, significantly enriched species in the HC group included [*Eubacterium*] *rectale*, *Oscillospiraceaebacterium*, *Faecalibacterium prausnitzii*, *Bifidobacterium adolescentis*, *Prevotella copri*, and *Blautia obeum*. By contrast, [*Ruminococcus*] *gnavus* and *Escherichia coli* were significantly enriched in the HTN group.

GO functional enrichment analysis was further performed to explore microbial functional variations (Fig. 2). In the Biological Process category, multiple pH homeostasis-related pathways were significantly downregulated in the HTN group, including cellular pH reduction, intracellular pH reduction, and lithium ion response, suggesting impaired capacity to maintain intracellular acid-base balance. Moreover, both positive and negative regulatory pathways of endoribonuclease activity were significantly suppressed, indicating impaired microbial RNA degradation and regulatory functions. Conversely, upregulated pathways in HTN patients mainly involved transcriptional regulation (DNA-binding transcription factor activity), alcohol stress responses (ethanol and alcohol response), and quinolinate metabolism and biosynthesis.

**Fig. 2.** Functional differences of gut microbiota between HTN patients and HC. The Wilcoxon rank-sum test was performed on the abundance matrix obtained from GO functional annotation to evaluate functional differences for each GO term between groups. The bar chart displays the top 20 most significantly enriched pathways in each category. Blue represents downregulated functions in the HTN group, while red indicates upregulated functions in the HTN group. HTN: Herpetic trigeminal neuralgia; HC: healthy controls; UV: Ultraviolet; GTPase: guanosine triphosphatase; ATP: adenosine triphosphate; NAD: nicotinamide adenine dinucleotide; NADP: nicotinamide adenine dinucleotide phosphate.

In the Cellular Component category, the aminodeoxychorismate synthase complex, pellicle, and inner membrane pellicle complex were significantly enriched in the HTN group. For Molecular Function, ribonuclease inhibitor activity exhibited the most prominent downregulation. Additional suppressed functions included nitrite transmembrane transporter activity, oxidoreductase activity, and hydrogen dehydrogenase nicotinamide adenine dinucleotide phosphate, oxidized form (NADP⁺) activity. In contrast, upregulated molecular functions in HTN group comprised kinase regulator activity and protein kinase regulator activity, as well as multiple metabolic enzyme activities, including acetylesterase, short-chain carboxylesterase, mannitol-1-phosphate 5-dehydrogenase, mannosyltransferase, and glutamate-cysteine ligase activities. These functional alterations were primarily associated with oxidative stress defense and polysaccharide biosynthesis and metabolism.

Collectively, the gut microbiota of HTN patients exhibited systematic downregulation of core homeostasis functions, including pH stabilization, nitrite metabolism, redox balance, and ribonuclease inhibition. Meanwhile, compensatory upregulation was observed in pathways related to guanosine triphosphatase (GTPase)/kinase-mediated signaling, polysaccharide metabolism, bacterial membrane structure remodeling, and oxidative stress responses. These findings indicate that gut microbial dysfunction may participate in HTN pathological progression through multiple biological pathways, although causal inference cannot be drawn from this observational study design.

### 3.2 HTN is associated with altered fecal metabolite profiles in patients

Volcano plot analysis of fecal metabolites revealed extensive metabolic perturbations in HTN patients (Fig. 3a). Compared with HC, the HTN group presented a greater number of significantly upregulated metabolites with larger fold changes, indicating profound disruption of the intestinal metabolic microenvironment in HTN patients.

To screen robust metabolite biomarkers, differentially abundant fecal metabolites identified via the limma package were subjected to LASSO regression analysis for dimensionality reduction and feature selection. A panel of 14 candidate fecal metabolites were finally retained (Fig. 3b). LASSO regression with binomial deviance cross-validation was further applied to determine core predictive variables and establish a predictive model. The area under the receiver operating characteristic (ROC) curve (AUC) for the model in the test set was 0.833, indicating favorable discriminatory power (**Supplementary Fig. 1a,b**).

**Fig. 3.** Identification and evaluation of fecal differential metabolites. (a) Differential metabolites between the HC and HTN groups were systematically screened using the limma linear model based on fecal metabolomic data. Raw data were transformed via log_2_(x+1) to satisfy the normality assumption. A design matrix without an intercept term was constructed, with the contrast set as HTN versus HC. Linear fitting was performed using the lmFit function, and intergroup comparisons were calculated via contrasts fit. Empirical Bayesian smoothing was implemented with the eBayes function to improve the stability of statistical testing. The Benjamini-Hochberg (BH) method was applied for false discovery rate (FDR) correction in multiple testing. Metabolites with (*p* < 0.05 and |log_2_FC| > 1) were defined as significantly differentially abundant metabolites. (b) All samples were randomly divided into a training set (n = 26) and a test set (n = 11) at a ratio of 7:3. Binomial logistic regression (family = “binomial”) was adopted as the modeling framework. Metabolites with non-zero coefficients were extracted as candidate diagnostic features, and their expression patterns were visualized by heatmap. HTN: Herpetic trigeminal neuralgia; HC: healthy controls.

Notably, several candidate metabolites corresponded to clinically administered drugs, including Ganciclovir, Paracetamol, and Metformin. Given the study design, the accumulation of drug-related metabolites likely reflected individual medication exposure histories rather than inherent HTN-specific metabolic alterations. Accordingly, these compounds were interpreted as medication-related signatures and excluded from disease-related biomarker interpretation. Additionally, multiple xanthine derivatives, such as 2′,7-Dihydroxy-4′-methoxy-8-prenylflavan and 8-[(2-Hydroxyethyl) amino]-1,3,7-trimethylxanthine, were significantly enriched in the HTN patients, implying that aberrant purine pathway activation may contribute to HTN progression. In contrast, 9,10,13-Trihydroxystearic acid and 4-(4-Fluoroanilino)-2H-chromen-2-one were predominant in HC, and their depletion in HTN patients suggested impaired intestinal lipid metabolic homeostasis under disease conditions.

### 3.3 HTN is associated with altered serum metabolite profiles in patients

Untargeted metabolomic profiling was further performed on serum samples to characterize systematic metabolic alterations in HTN patients. In contrast to the fecal metabolome, the serum metabolome exhibited relatively mild perturbations, with fewer significantly differentially abundant metabolites identified between the two groups (Fig. 4a).

Among the differential serum metabolites identified (Fig. 4b), Metoprolol, 2,2′-(4-(2-Hydroxyethylamino)-3-nitrophenylazanediyl)diethanol, dimethylidenebutanedioylcarnitine, Trigonelline, 3-Pyridylacetic acid, and Aurintricarboxylic acid were significantly elevated in HTN patients, whereas 3,3,4,4,5,5,6,6,6-Nonafluorohexyl 2-methylacrylate, Trifluoroacetic acid, and Euparin were significantly decreased. Notably, Metoprolol—a widely prescribed β-adrenergic receptor blocker—was detected at elevated levels in patient serum. This enrichment was attributed to clinical medication exposure rather than inherent HTN-associated metabolic dysregulation, thereby representing a medication-related signature instead of a disease-specific biomarker. Of note, both Trigonelline and 3-Pyridylacetic acid are key intermediates involved in nicotinate metabolism. Additionally, Euparin, a naturally coumarin derivative with documented anti-inflammatory and neuroprotective properties, was significantly downregulated in HTN patients, suggesting its potential protective role in HTN pathogenesis.

Subsequent KEGG pathway enrichment analysis was performed based on all differential serum metabolites (*p* < 0.05). The results indicated significant enrichment of the nicotinate and nicotinamide metabolism pathway and the retrograde endocannabinoid signaling pathway in the HTN group (Fig. 4c), implying that dysregulation of these two metabolic signaling axes may contribute to HTN progression.

**Fig. 4.** Identification of serum differential metabolites. (a) The limma linear model was similarly applied to analyze differential metabolites between the HC and HTN groups. With the screening thresholds set at *p* < 0.05 and |log2FC| > 1, a total of nine characteristic differential metabolites were identified. (b) Heatmap of serum differential metabolites. (c) Metabolite pathway Sankey diagram-enrichment Bubble Plot. HTN: Herpetic trigeminal neuralgia; HC: healthy controls; PC: Phosphatidylcholine.

### 3.4 Correlations between differential gut microbiota and metabolites

To systematically explore the potential regulatory associations between differential gut microbial taxa and fecal metabolites in HTN patients, random forest regression models were constructed. Differential microbial species and genera were incorporated as independent variables to predict the abundance variation of candidate fecal metabolites, thereby elucidating microbe–metabolite interaction patterns (Fig. 5).

**Fig. 5.** Contribution of characteristic microorganisms to changes in fecal signature metabolites. The prediction model was constructed using the randomForest package, with the number of decision trees set to 500 (ntree = 500) and variable importance evaluation enabled (importance = TRUE). All target variables were ranked in descending order according to their total feature importance scores, and the top 20 variables with the highest contribution were screened to plot a stacked bar chart. The length and corresponding value of each bar represent the influence level of alterations in characteristic microorganisms on changes in fecal signature metabolites.

The model analysis identified 2,6-Xylidine as the fecal metabolite most strongly modulated by gut microbiota. *Lachnospira*, *Clostridium innocuum*, *Lachnospira eligens*, *Simiaoa sunii*, and *Roseburia hominis* were recognized as the potential microbial contributors influencing its abundance.

Among medication-related metabolites, Metformin exhibited prominent correlations with gut microbiota composition. Specifically, *Haemophilus parainfluenzae*, *Haemophilus*, *Veillonella parvula*, *Eubacterium*, and *Erysipelatoclostridium* showed strong interactive relationships with Metformin, indicating that these intestinal metabolites of Metformin may be significantly modulated by these microbial taxa.

Furthermore, 2-Hydroxy-2-(propan-2-yl) butanedioylcarnitine exhibited feature importance scores >7 for *Enterocloster bolteae*, *Eggerthella*, and *Eggerthella lenta*, suggesting substantial microbial contributions to the regulation of this carnitine-related metabolite. In addition, 9,10,13-Trihydroxystearic acid was strongly associated with *Wujia* and *Wujia chipingensis* (importance score >7), suggesting that the abundance of this hydroxylated fatty acid is influenced by these newly identified microbial taxa.

Collectively, the exploratory random forest regression analysis revealed correlations between differential gut microbiota and fecal metabolites in the HTN cohort, providing novel clues for understanding the microbe-mediated metabolic dysregulation underlying HTN pathological progression.

WGCNA was further conducted to integrate fecal and serum metabolite profiles, aiming to systematically characterize coordinated cross-compartment metabolic perturbation patterns underlying HTN pathogenesis and reveal coordinated metabolic alteration patterns between the two compartments in the context of HTN (Fig. 6).

**Fig. 6.** Weighted gene co-expression network analysis (WGCNA) was performed for integrated modular analysis of serum and fecal metabolites. A total of 200 metabolites were included, consisting of 50 serum differential metabolites and 150 fecal differential metabolites with the largest variance. The analyzed samples comprised the HC group (n = 18) and the HTN group (n = 19).

A total of four metabolite co-expression modules were identified in the constructed network, including the turquoise module (86 metabolites), blue module (51 metabolites), brown module (44 metabolites), and grey module (19 unclassified metabolites). Pearson correlation analysis between MEs and the HTN phenotype status was subsequently performed to evaluate the phenotypic relevance of each metabolic module.

The blue module (*r* = 0.73, *p* = 3.8 × 10^−7^) and the brown module (*r* = 0.73, *p* = 3.3 × 10^−7^) were both found to be significantly positively correlated with the HTN phenotype, indicating that the metabolites comprising these two modules were globally upregulated in HTN patients. In contrast, although the turquoise module contained the largest number of metabolites, its correlation with the HTN phenotype did not reach statistical significance (*r* = 0.14, *p* = 0.19). Furthermore, the distribution of GS-HTN values within the turquoise module were predominantly negative, suggesting that the majority of metabolites in this module were downregulated in both feces and serum samples from HTN patients. These observations imply that the progression of HTN is accompanied by a widespread suppression of systemic metabolic activity.

Based on the GS–MM scatter plots, metabolites with currently high MM and high GS values were prioritized as candidate key metabolites within each module.

In the blue module, the fecal-derived metabolites 3-{[(1Z)-4-(4-Hydroxyphenyl) phthalazin-1(2H)-ylidene] amino} phenol and 2-(4-Pyridinyl)-4-quinolinecarbohydrazide both exhibited MM values >0.8 and GS values >0.3, indicating strong intramodular connectivity and robust association with the HTN phenotype. These metabolites were therefore designated as potential core metabolites of the blue module (**Supplementary Fig. 2a**, **Supplementary Table 1**).

In the brown module, multiple fecal-derived metabolites, including 5,7-Dihydroxy-2-(4-methoxyphenyl)-6-(3-methylbut-2-enyl)-2,3-dihydrochromen-4-one, 1,9-Dideoxyforskolin, Homoorientin, N-Acetyl-β-alanine, 7-[2,6-dimethyl-8-(2-methylbutanoyloxy)]heptanoic acid, Benzoic acid, Benzyl 6-O-(methylbutanoyl)-β-D-glucopyranoside, Isogravacridonechlorine, and Austalide L, along with serum-derived metabolites including Glycodeoxycholic acid, Glycochenodeoxycholic acid, and Glycohyodeoxycholic acid, all exhibited MM values >0.5 and GS values >0.3. These results suggested that the above metabolites were strongly correlated with both the brown module and the HTN phenotype. Notably, the clustered enrichment of conjugated bile acid metabolites implied that the bile acid metabolic axis may play an important role in the pathological progression of HTN (**Supplementary Fig. 2b**, **Supplementary Table 1**).

Hub metabolites within each module were subsequently identified based on connectivity values derived from eigengene analysis (kME (connectivity to the module eigengene)). The hub metabolite of the blue module was 3-{[(1Z)-4-(4-Hydroxyphenyl)phthalazin-1(2H)-ylidene]amino}phenol (kME = 0.83), whereas that of the brown module was 5,7-Dihydroxy-2-(4-methoxyphenyl)-6-(3-methylbut-2-enyl)-2,3-dihydrochromen-4-one (kME = 0.81). In the turquoise module, Cauloside C was designated as the hub metabolite (kME = 0.77). All three hub metabolites exhibited kME values >0.76, indicating a high degree of consistency with the co-expression patterns of metabolites within their respective modules (**Supplementary Table 1**).

To further evaluate the stability of module classification, bootstrap analysis was conducted by repeatedly reconstructing the co-expression network 100 times, each time using a randomly selected 80% subset of the samples. Module preservation analysis yielded a stability score of 0.840 ± 0.000 within the current dataset, indicating that the identified module structures were reasonably reproducible and relatively stable. These findings thus provide a preliminary basis for subsequent functional analyses (**Supplementary Fig. 2c**).

### 3.5 Association analysis of characteristic microorganisms and metabolites with HTN disease-related clinical features

To validate the associations between the identified candidate features and clinical pain characteristics, the VAS scores were used as a quantitative indicator of pain severity (Fig. 7).

**Fig. 7.** Correlations between metabolite/microbial markers and pain scores. (a) Correlation fitting curves between each metabolite marker and pain score using individually matched patient samples, with *p* < 0.05 set as the threshold for statistical significance. (b) Correlation fitting curves between each microbial marker and pain score in patient samples. VAS: Visual Analogue Scale.

Most characteristic metabolites were found to be negatively correlated with VAS scores (Fig. 7a). Among the medication-related metabolites, Ganciclovir (*r* = −0.446) and 8-Cyclopentyl-1,3-dimethylxanthine (*r* = −0.546) both exhibited significant negative correlations with VAS scores, with the latter showing a stronger association. Additionally, 2′,7-Dihydroxy-4′-methoxy-8-prenylflavan-2′,7-diglucoside also demonstrated a relatively strong negative correlation (*r* = −0.54), suggesting that the intestinal abundance of this flavonoid compound may be closely associated with pain alleviation. Notably, 4-(4-Fluoroanilino)-2H-chromen-2-one was the only characteristic metabolite that showed a positive correlation with VAS scores (*r* = 0.475), implying that its abundance increased with greater pain severity.

Correlation analysis between characteristic fecal microorganisms and VAS scores further revealed distinct association patterns among different microbial genera (Fig. 7b). *Haemophilus*, *Roseburia*, *Simiaoa*, *Lachnospira*, *Faecalibacterium*, and *Anaerobutyricum* were found to be significantly positively correlated with VAS scores, suggesting that the abundances of these genera increased with higher pain scores and may reflect reactive or adaptive responses during disease progression. In contrast, *Eggerthella*, *Erysipelatoclostridium*, *Enterocloster*, and *Mediterraneibacter* were significantly negatively correlated with VAS scores, indicating that a decrease in these genera was associated with aggravated pain severity. These microorganisms may, therefore, be involved in relieving HTN-related pain, potentially through anti-inflammatory activity or neuromodulatory metabolic pathways.

Among all microbial taxa analyzed, *Haemophilus*, *Eggerthella*, and *Erysipelatoclostridium* exhibited the strongest correlations with VAS scores, suggesting their potential utility as candidate microbial features indicative of pain severity in HTN patients.

## 4. Discussion

This study systematically investigated gut microbial perturbations and candidate markers in patients with HTN. Accumulating evidence has validated bidirectional signaling crosstalk between the gut microbiota and the nervous system, which is mediated by vagal nerve pathways, immune regulatory mediators, microbiota-derived small-molecule metabolites, and peripheral circulatory signals. As a core immunoregulatory system in humans, the gut microbiota profoundly modulates neural function via intricate metabolic cascades and signaling networks. Specifically, gut microbiota-associated mechanisms include the regulation of BBB permeability, participation in the pathological progression of neurodegenerative disorder, and mediation of inter-axis communication between microbial extracellular vesicles and neuroepithelial cells [4, 5, 15].

Based on the theoretical basis outlined above, the present study further characterizes the ecological profiles of the gut–brain axis and its underlying metabolic regulatory mechanisms in HTN patients. Fecal samples from HTN patients and samples from HC were subjected to metagenomic sequencing and untargeted metabolomic analyses. Meanwhile, untargeted metabolomics profiling of serum samples from the same cohort was performed to elucidate alterations in gut microbial composition, microbiota-mediated intestinal metabolic dysregulation, and their intrinsic correlations with peripheral serum metabolic signatures in HTN patients. Integrating multiomics analysis constructed a multilayered interaction framework of gut–brain axis, namely the “gut microbiota–intestinal metabolism–peripheral circulation” axis. Collectively, this study provides preliminary correlational evidence for the neuroimmune–metabolic mechanisms underlying HTN pathogenesis, which can facilitate future hypothesis-driven studies of HTN associated neuroimmune and metabolic perturbations.

The human gut represents a highly dynamic microecosystem harboring over 250 species of bacteria, archaea, fungi, and viruses, with its microbial composition continuously remodeling throughout the host lifespan. Under physiological conditions, the gut microbiota is predominated by five core bacterial phyla, including Firmicutes (approximately 60%–80%, mainly consisting of Clostridia and Bacilli), Bacteroidetes (20%–40%), Verrucomicrobia, Actinobacteria, and Proteobacteria, along with a small proportion of archaea [16]. These microbial communities produce abundant functional metabolites, such as short-chain fatty acids (SCFAs), via intricate enzymatic reactions, and thereby maintaining intestinal homeostasis and modulating host immune function. In contrast, pathological stimuli can trigger the overproduction of inflammatory cytokines by specific microbial products, which further induce intestinal epithelial injury and sustain chronic inflammatory progression [17].

Consistently, the present study identified distinct gut microbiota dysbiosis in HTN patients, characterized by significant enrichment of Proteobacteria and obvious depletion of Firmicutes relative to HC. This microbial alteration is highly congruent with findings from previous studies on gut microbial perturbations in postherpetic neuralgia and primary headache disorders, both of which have documented reduced Firmicutes abundance in diseased populations [8]. Previous studies have shown that Firmicutes abundance is negatively correlated with the level of pro-inflammatory cytokines and toxic metabolites, while positively associated with the production of beneficial metabolites including SCFAs [18]. Pathogenic microbes exhibit superior proliferative capacity under immune-deficient conditions and can adapt to and utilize metabolites derived from inflammatory microenvironments to facilitate their own proliferation. This process establishes a self-perpetuating vicious cycle of “inflammation-induced dysbiosis, dysbiosis-amplified inflammation, and metabolic disorder”, which may serve as a pivotal microecological mechanism driving the persistent progression of HTN.

At the genus level, *Mediterraneibacter* and *Escherichia* were significantly enriched in the HTN group. At the species level, [*Ruminococcus*] *gnavus* and *Escherichia coli* were markedly accumulated in HTN patients. [*Ruminococcus*] *gnavus* is a commensal human gut bacterium closely associated with the development and progression of multiple diseases. Previous studies have confirmed that its abundance is significantly elevated in patients with active inflammatory bowel disease, and its enrichment level is positively correlated with disease severity. These findings indicate that this bacterium can not only exacerbate intestinal inflammation, but also achieve selective proliferation in inflammatory microenvironments [19]. Mechanistically, functional investigation in inflammatory bowel disease has validated that [*Ruminococcus*] *gnavus* secretes a structurally complex glucorhamnan polysaccharide, which potently stimulates dendritic cells to secrete the pro-inflammatory cytokine tumor necrosis factor-α and further amplifies intestinal inflammatory cascades [20].

*E. coli* normally resides at relatively low abundance in the healthy gut microbiota, whereas its aberrant expansion may trigger systemic neuroinflammatory responses. Existing studies have illustrated that *Escherichia coli* infection induces the secretion of casein kinase 2 (CK2) by brain microvascular endothelial cells. The β-subunit of CK2 further interacts with astrocytic myoglobin, subsequently activating the downstream Nuclear Factor kappa-B (NF-κB) signaling pathways and initiating early inflammatory responses in astrocytes. Collectively, these results reveal a plausible mechanism whereby elevated intestinal *E. coli* abundance contributes to central nervous system inflammation via signaling crosstalk at the neurovascular interface [21]. GO enrichment analysis of characteristic microbial taxa revealed significant downregulation of core functional pathways in the gut microbiota of HTN patients, including pH regulation, nitrite metabolism, redox activity, and ribonuclease inhibition.

Previous studies have confirmed that the gut microbiota modulates intestinal pH microenvironments by regulating the activity of acid-producing bacteria through nitrite metabolic cascades [22]. Dysregulated nitrite metabolism disrupts gut redox balance, destabilizes the intestinal ecosystem oxidative–reductive equilibrium, and further triggers aberrant proliferation of acid-producing bacteria, altered microbial fermentation profiles, and progressive aggravation of gut dysbiosis [23].

These functional perturbations are highly consistent with the structural microbial dysbiosis observed in HTN patients, indicating that gut microbial dysfunction contributes to HTN pathogenesis through a multi-dimensional metabolic–inflammatory–neurological regulatory network. Intestinal pH homeostasis is predominantly maintained by SCFAs, including acetate, propionate, and butyrate. As pivotal metabolites derived from bacterial carbohydrate fermentation, SCFAs are indispensable for maintaining intestinal barrier integrity and immune homeostasis [22]. Among these metabolites, butyrate is one of the most important SCFAs in humans, with its neuroprotective efficacy preliminarily validated in multiple clinical investigations.

For instance, interventional studies in Parkinson’s disease have demonstrated that oral resistant starch supplementation increased intestinal SCFA and butyrate levels, accompanied by remarkable improvement in disease-related non-motor symptoms [24]. In addition, meta-analyses of neurological disorders (*e.g.*, myalgic encephalomyelitis) have verified that disease progression is tightly correlated with the depletion of butyrate-producing bacteria [25], which is consistent with the microbial dysbiosis profile characterized in HTN patients in the present study.

In terms of redox function, previous studies on postherpetic neuralgia have reported significantly decreased levels of total thiols and native thiols, indicative of excessive oxidative stress and impaired systemic antioxidant defense in neuropathological states [26]. These findings corroborate the suppressed redox-related microbial pathways identified via GO functional enrichment analysis in the current HTN cohort.

Pairwise correlation analysis between characteristic metabolites and VAS pain scores identified 8-Cyclopentyl-1,3-dimethylxanthine (8-CPT) as the metabolite with the strongest negative correlation with pain severity. Notably, 8-CPT is an exogenous compound originating from medication rather than endogenous metabolic synthesis. Its detection in HTN patients is attributable to medication history, and its negative correlation with pain intensity does not qualify it as an endogenous HTN biomarker. Instead, 8-CPT represents a medication-exposure-specific signature, and its correlation with pain scores likely reflects the pharmacological analgesic effects of clinical interventions.

Serum metabolomic differential analysis screened Aurintricarboxylic acid (ATA) as a significantly upregulated metabolite in HTN patients. ATA is a multifunctional bioactive molecule that potently antagonizes P2X3 receptor-mediated signaling at nanomolar concentrations [27] and modulates multiple core inflammatory and cell survival pathways, including the TNF-related weak inducer of apoptosis/Fibroblast growth factor-inducible 14 (TWEAK/Fn14) axis, NF-κB/p65 signaling pathway, and the Transcriptional coactivator with PDZ-binding motif–TEA domain transcription factor (TAZ–TEAD) transcriptional regulation [28].

P2X purinoceptor 3 (P2X3) receptors are widely expressed in primary sensory neurons of the trigeminal ganglion and serve as core ion channels mediating nociceptive signal transduction [29]. Accordingly, ATA accumulation in HTN patients may constitute an endogenous protective feedback response triggered by persistent trigeminal nerve injury, which alleviates excessive nociceptive signaling via P2X3 receptor antagonism.

Pathway enrichment analysis further identified two significantly perturbed metabolic signaling pathways in HTN patients: nicotinate and nicotinamide metabolism, and retrograde endocannabinoid signaling.

The primary differential metabolites enriched in the nicotinate and nicotinamide metabolic pathway included N1-Methyl-2-pyridone-5-carboxamide, 1-Methylnicotinamide, and Nicotinamide. Nicotinamide exerts prominent anti-neuroinflammatory effects under neuropathological conditions through nicotinamide adenine dinucleotide, oxidized form (NAD⁺)-dependent deacetylation targeting the NF-κB p65 subunit. Nicotinamide supplementation has been proven to elevate cerebral NAD⁺ levels and effectively suppress microglial activation [30].

NAD⁺ homeostasis is dynamically maintained by the balance between synthesis and catabolic pathways, with tryptophan and nicotinamide serving as its primary biosynthetic precursors. Accumulating evidence indicates that NAD⁺ depletion acts as a pivotal pathogenic driver of multiple hereditary and acquired disorders [31]. Collectively, these observations suggest that disrupted nicotinamide metabolism in HTN patients impairs cerebral NAD⁺ homeostasis, thereby exacerbating neuroinflammation and neuronal injury.

Two differential lipid metabolites were enriched in the retrograde endocannabinoid signaling pathway in HTN patients: 1-(1Z-Octadecenyl)-2-(5Z,8Z,11Z,14Z-eicosatetraenoyl)-sn-glycero-3-phosphoethanolamine and Phosphatidylcholine (PC) (16:0/20:4). Previous studies using mouse models of peripheral nerve injury demonstrated that PC (16:0/20:4) was significantly increased in the spinal cord following sciatic nerve transection. Its spatiotemporal distribution was closely associated with reactive microglia and astrocytes, which are key regulators of neuroinflammatory responses [32]. These findings provide solid mechanistic evidence supporting the neuro–immune–metabolic crosstalk observed in the present study.

Overall, the initiation and progression of HTN involve coordinated systemic responses of multiple host metabolites. Microbiota-derived small molecules modulate multi-level signaling along the gut–brain axis, synergistically regulating disease pathogenesis. This novel multidimensional metabolic–neurological–immunological interaction model advances the current understanding of HTN pathological mechanisms.

WGCNA revealed that all hub metabolites in the three identified co-expression modules were of fecal origin, highlighting the central coordinating role of intestinal metabolism in cross-tissue metabolite co-expression network. Fecal metabolic perturbations may drive systematic alterations in peripheral serum metabolic profiles via gut–blood axis signaling, further validating the core regulatory function of intestinal metabolism in shaping systemic metabolic homeostasis at the network level.

Random forest regression analysis was performed to screen key microbial taxa correlated with characteristic fecal metabolites. Among all differential metabolites, 2,6-Xylidine exhibited the strongest association with gut microbial composition and was primarily correlated with *Lachnospira*. *Lachnospira* is a beneficial gut microbe critical for maintaining intestinal barrier integrity and regulating host immune responses [33], and its reduced abundance has been closely linked to the progression of multiple metabolic and inflammatory diseases, including type 2 diabetes mellitus, IBD, and obesity, as well as several malignancies [34, 35].

As another core butyrate-producing genus, *Roseburia*, together with *Lachnospira* eligens and *Roseburiahominis*, constitutes the dominant microbial consortium modulating intestinal 2,6-Xylidine levels [36]. The specific correlation between butyrate-producing bacteria and aromatic amine metabolites indicates that intestinal concentrations of aromatic compounds, such as 2,6-Xylidine, are directly regulated by the abundance of beneficial butyrate-producing microbes. Deficits in these protective microbial taxa can further trigger cascading disturbances in systemic downstream metabolic profiles.

Of note, *Wujia* and *Wujia chipingensis* are newly characterized bacterial taxa in human gut microecological research. Existing studies have reported their enrichment in patients with traumatic brain injury, with their abundance negatively correlated with C-reactive protein, implying potential neuroprotective and anti-inflammatory functions [37]. In the present study, *Wujia* and *Wujia chipingensis* showed strong correlations with 9,10,13-Trihydroxystearic acid, further linking gut microbial remodeling and metabolic dysfunction to HTN pathogenesis. Therefore, gut microbiota-targeted intervention is a promising therapeutic direction for HTN; however, the clinical efficacy of such strategies requires further validation in prospective randomized controlled trials.

Correlation analysis between metabolites and pain severity identified 4-(4-Fluoroanilino)-2H-chromen-2-one as the only characteristic metabolite positively correlated with VAS scores. Nevertheless, this compound is speculated to be a pharmaceutical intermediate or medication metabolite rather than an endogenous pathogenic molecule. The gut microbiota is a critical regulator of intestinal drug biotransformation, and microbial metabolic activity profoundly affects systemic drug bioavailability and exposure levels [38]. Accordingly, the accumulation of 4-(4-fluoroanilino)-2H-chromen-2-one in HTN patients primarily reflects disordered medication metabolism induced by gut microbial dysbiosis, rather than a direct pain-promoting biological effect. This compound should, thus, be interpreted as a medication-exposure-associated signature instead of a disease-specific biomarker for HTN.

In summary, this study integrated metagenomic, fecal metabolomic, and serum metabolomic datasets to systematically characterize the gut microbial and metabolic landscape of HTN patients. HTN patients exhibited typical gut dysbiosis characterized by enriched *Proteobacteria*, depleted *Firmicutes*, reduced butyrate-producing bacteria, and expanded opportunistic pathogens. Structurally altered microbiota was accompanied by functional suppression of core homeostatic pathways, including intestinal pH regulation and redox defense systems.

At the metabolic level, exogenous 8-CPT may modulate endogenous analgesic signaling via adenosine A1 receptor antagonism, while ATA upregulation represents an endogenous neuroprotective feedback response against persistent neural injury. Dysregulated nicotinamide metabolism and impaired retrograde endocannabinoid signaling further contribute to chronic neuroinflammation and sustained pain in HTN patients. WGCNA confirmed that fecal metabolites act as core regulatory nodes in the gut–blood metabolic network, and random forest regression analysis further elucidated the quantitative correlations between key microbial taxa and fecal metabolites. Integrative metabolite–VAS correlation analyses further bridge molecular dysregulations and clinical pain phenotypes, providing candidate translational biomarkers for HTN. Further independent validation in larger clinical cohorts is required to verify the stability and efficacy of candidate features.

## 5. Limitations

This study has several inherent limitations attributable to its cross-sectional, exploratory design and relatively small sample size, which prevented the comprehensive control of all potential confounding factors in the analytical models. Disease duration was recorded for patients and was not applicable to HC, making it unavailable for intergroup matching. Given that disease duration is a well-recognized factor shaping gut microbiota composition and metabolite profiles, it may serve as an unadjusted confounder in the present analysis. However, the small sample size of the HTN group limited our ability to perform robust stratified analyses or multivariable adjustment according to disease duration.

In addition, detailed medication histories were not systematically collected per the original study protocol. This limitation hinders the formal statistical adjustment for confounding effects induced by concomitant medications, including antihypertensive, hypoglycemic, and analgesic agents. Medication-related interference may alter host metabolomic profiles, which suggests that the differential metabolites identified in this study should be interpreted with caution. Such confounding effects may compromise the specificity of these metabolites as candidate biomarkers for HTN. Moreover, this retrospective cohort lacked complete data on multiple clinically critical variables, including dietary habits, rash conditions, herpes zoster treatment history, comorbidities, and detailed medication exposure.

Notably, this is a single-center study conducted in China, where the study population may be subject to unique geographical characteristics and dietary patterns, potentially limiting the generalizability of the findings. Future studies based on large-scale, multicenter, and prospective cohorts with standardized and comprehensive collection of disease duration, medication exposure, dietary information, and other clinical covariates are, therefore, required to rigorously verify and eliminate potential confounding biases.

## 6. Conclusions

This cross-sectional study confirms significant gut microbiota dysbiosis in patients with HTN. Microbial alterations in HTN patients are characterized by the enrichment of *Proteobacteria* and opportunistic pathogens (*e.g.*, *Escherichia coli* and [*Ruminococcus*] *gnavus*), as well as the depletion of beneficial Firmicutes and butyrate-producing bacteria, including *Lachnospira* and *Roseburia*. Functionally, these microbial perturbations are accompanied by the downregulated activities of intestinal pH regulation, nitrite metabolism, and redox defense pathways. These changes collectively disrupt intestinal homeostasis and sustain a vicious cycle of inflammation-mediated gut microbiota dysbiosis.

Integrated fecal and serum metabolomic profiling further identified a panel of differential metabolites closely correlated with gut microbial dysfunction and systemic metabolic disorders in HTN, including aberrant nicotinamide metabolism and impaired endocannabinoid signaling. Correlation analysis with VAS pain scores demonstrated that 9,10,13-Trihydroxystearic acid, a metabolite associated with the newly identified taxa *Wujia* and *Wujia chipingensis*, is significantly linked to pain-related microbial variations, indicating its potential as a promising candidate biomarker for pain evaluation in HTN patients. Collectively, these exploratory multi-omics findings provide preliminary evidence for the involvement of the gut–brain axis in the pathophysiology of HTN, from the integrated perspective of “gut microbiota–intestinal metabolism–peripheral circulation–neuroinflammation”. This study lays a theoretical foundation for in-depth mechanistic research on HTN. Nevertheless, the clinical translational value of the present findings requires rigorous validation in large, prospective, multicenter cohorts.

## Supplementary Material

Supplementary material associated with this article can be found, in the online version, at https://files.jofph.com/files/article/2098303667524255744/attachment/Supplementary%20material.zip.





## References

[1] Yawn BP, Gilden D. The global epidemiology of herpes zoster. Neurology. 2013; 81: 928–930. 10.1212/WNL.0b013e3182a3516e PMC3885217 23999562

[2] Wu C, Lv Y, Wang C, Zhong C. Efficacy of semilunar ganglion radiofrequency thermal coagulation in moderate and severe trigeminal postherpetic neuralgia and its impact on serum IL-1β and IL-6 levels. European Journal of Medical Research. 2025; 30: 1005. 10.1186/s40001-025-03257-0 PMC12542188 41121439

[3] Niemeyer CS, Harlander-Locke M, Bubak AN, Rzasa-Lynn R, Birlea M. Trigeminal postherpetic neuralgia: from pathophysiology to treatment. Current Pain and Headache Reports. 2024; 28: 295–306. 10.1007/s11916-023-01209-z PMC10940365 38261232

[4] Braniste V, Al-Asmakh M, Kowal C, Anuar F, Abbaspour A, Tóth M, *et al*. The gut microbiota influences blood-brain barrier permeability in mice. Science Translational Medicine. 2014; 6: 263ra158. Erratum in: Science Translational Medicine. 2014; 6: 266er7. 10.1126/scitranslmed.3009759 PMC4396848 25411471

[5] Hassan A, Huang Y, Mahjabina, Sudiro OH, Wang X, He J, *et al*. Emerging role of gut microbiota extracellular vesicle in neurodegenerative disorders and insights on their therapeutic management. iMetaOmics. 2024; 1: e33. 10.1002/imo2.33 PMC12806478 41676116

[6] Sampson TR, Debelius JW, Thron T, Janssen S, Shastri GG, Ilhan ZE, *et al*. Gut microbiota regulate motor deficits and neuroinflammation in a model of Parkinson’s disease. Cell. 2016; 167: 1469–1480.e12. 10.1016/j.cell.2016.11.018 PMC5718049 27912057

[7] Pluta R, Ułamek-Kozioł M, Januszewski S, Czuczwar SJ. Gut microbiota and pro/prebiotics in Alzheimer’s disease. Aging. 2020; 12: 5539–5550. 10.18632/aging.102930 PMC7138569 32191919

[8] Jiao B, Cao X, Zhang C, Zhang W, Yu S, Zhang M, *et al*. Alterations of the gut microbiota in patients with postherpetic neuralgia. AMB Express. 2023; 13: 108. 10.1186/s13568-023-01614-y PMC10558420 37803181

[9] Headache Classification Committee of the International Headache Society (IHS) The International Classification of Headache Disorders, 3rd edition. Cephalalgia. 2018; 38: 1–211. 10.1177/0333102417738202 29368949

[10] Wei R, Wang J, Su M, Jia E, Chen S, Chen T, *et al*. Missing value imputation approach for mass spectrometry-based metabolomics data. Scientific Reports. 2018; 8: 663. 10.1038/s41598-017-19120-0 PMC5766532 29330539

[11] Sumner LW, Amberg A, Barrett D, Beale MH, Beger R, Daykin CA, *et al*. Proposed minimum reporting standards for chemical analysis chemical analysis working group (CAWG) metabolomics standards initiative (MSI). Metabolomics. 2007; 3: 211–221. 10.1007/s11306-007-0082-2 PMC3772505 24039616

[12] Conesa A, Götz S, García-Gómez JM, Terol J, Talón M, Robles M. Blast2GO: a universal tool for annotation, visualization and analysis in functional genomics research. Bioinformatics. 2005; 21: 3674–3676. 10.1093/bioinformatics/bti610 16081474

[13] Ashburner M, Ball CA, Blake JA, Botstein D, Butler H, Cherry JM, *et al*. Gene ontology: tool for the unification of biology. The Gene Ontology Consortium. Nature Genetics. 2000; 25: 25–29. 10.1038/75556 PMC3037419 10802651

[14] Zhang B, Horvath S. A general framework for weighted gene co-expression network analysis. Statistical Applications in Genetics and Molecular Biology. 2005; 4: Article17. 10.2202/1544-6115.1128 16646834

[15] Ohara TE, Hsiao EY. Microbiota-neuroepithelial signalling across the gut-brain axis. Nature Reviews Microbiology. 2025; 23: 371–384. 10.1038/s41579-024-01136-9 39743581

[16] Di Vincenzo F, Del Gaudio A, Petito V, Lopetuso LR, Scaldaferri F. Gut microbiota, intestinal permeability, and systemic inflammation: a narrative review. Internal and Emergency Medicine. 2024; 19: 275–293. 10.1007/s11739-023-03374-w PMC10954893 37505311

[17] Karczewski J, Poniedziałek B, Adamski Z, Rzymski P. The effects of the microbiota on the host immune system. Autoimmunity. 2014; 47: 494–504. 10.3109/08916934.2014.938322 25019177

[18] Bhat MI, Kapila R. Dietary metabolites derived from gut microbiota: critical modulators of epigenetic changes in mammals. Nutrition Reviews. 2017; 75: 374–389. 10.1093/nutrit/nux001 28444216

[19] Obana N, Nakato G, Nomura N, Fukuda S. A genetic toolkit for the human gut bacterium Mediterraneibacter gnavus identifies capsular polysaccharides as a competitive colonization factor. Nature Communications. 2026; 17: 3855. 10.1038/s41467-026-69022-x PMC13121745 41820342

[20] Henke MT, Kenny DJ, Cassilly CD, Vlamakis H, Xavier RJ, Clardy J. Ruminococcus gnavus, a member of the human gut microbiome associated with Crohn’s disease, produces an inflammatory polysaccharide. Proceedings of the National Academy of Sciences of the United States of America. 2019; 116: 12672–12677. 10.1073/pnas.1904099116 PMC6601261 31182571

[21] Huo D, Yang R, Fu J, Chen J, Tan C, Chen H, *et al*. CK2 derived from brain microvascular endothelial cells induces astrocyte inflammatory response in Escherichia coli-induced meningitis. PLOS Pathogens. 2025; 21: e1013464. 10.1371/journal.ppat.1013464 PMC12422478 40929057

[22] Crost EH, Coletto E, Bell A, Juge N. Ruminococcus gnavus: friend or foe for human health. FEMS Microbiology Reviews. 2023; 47: fuad014. 10.1093/femsre/fuad014 PMC10112845 37015876

[23] Tiso M, Schechter AN. Nitrate reduction to nitrite, nitric oxide and ammonia by gut bacteria under physiological conditions. PLOS ONE. 2015; 10: e0119712. Erratum in: PLOS ONE. 2015; 10: e0127490. 10.1371/journal.pone.0119712 PMC4372352 25803049

[24] Becker A, Schmartz GP, Gröger L, Grammes N, Galata V, Philippeit H, *et al*. Effects of resistant starch on symptoms, fecal markers, and gut microbiota in parkinson’s disease—the RESISTA-PD trial. Genomics, Proteomics & Bioinformatics. 2022; 20: 274–287. 10.1016/j.gpb.2021.08.009 PMC9684155 34839011

[25] Jurek JM, Castro-Marrero J. A narrative review on gut microbiome disturbances and microbial preparations in myalgic encephalomyelitis/chronic fatigue syndrome: implications for long COVID. Nutrients. 2024; 16: 1545. 10.3390/nu16111545 PMC11173566 38892479

[26] Arican S, Hacibeyoglu G, Ulukaya SO, Avcioglu G, Reisli R, Uzun ST, *et al*. Ischemia-modified albumin (IMA) and dynamic thiol-disulfide homeostasis in patients with postherpetic neuralgia. Journal of Laboratory Medicine. 2019; 43: 257–263.

[27] Obrecht AS, Urban N, Schaefer M, Röse A, Kless A, Meents JE, *et al*. Identification of aurintricarboxylic acid as a potent allosteric antagonist of P2X1 and P2X3 receptors. Neuropharmacology. 2019; 158: 107749. 10.1016/j.neuropharm.2019.107749 31461640

[28] Burman P, Jaiswal R, Devi K, Moharana B. Aurintricarboxylic acid protects isoproterenol induced left ventricular hypertrophy by modulating TWEAK signaling. Cardiovascular Pathology. 2022; 61: 107468. 10.1016/j.carpath.2022.107468 35977688

[29] Wijayanti IAS, Adnyana IMO, Widyadharma IPE, Wiratnaya IGE, Mahadewa TGB, Astawa INM. Neuroinflammation mechanism underlying neuropathic pain: the role of mesenchymal stem cell in neuroglia. AIMS Neuroscience. 2024; 11: 226–243. 10.3934/Neuroscience.2024015 PMC11486618 39431272

[30] Torres-Méndez JK, Niño-Narvión J, Martinez-Santos P, Diarte-Añazco EMG, Méndez-Lara KA, Del Olmo TV, *et al*. Nicotinamide prevents diabetic brain inflammation via NAD+-dependent deacetylation mechanisms. Nutrients. 2023; 15: 3083. 10.3390/nu15143083 PMC10383777 37513501

[31] Katsyuba E, Romani M, Hofer D, Auwerx J. NAD+ homeostasis in health and disease. Nature Metabolism. 2020; 2: 9–31. 10.1038/s42255-019-0161-5 32694684

[32] Xu D, Omura T, Masaki N, Arima H, Banno T, Okamoto A, *et al*. Increased arachidonic acid-containing phosphatidylcholine is associated with reactive microglia and astrocytes in the spinal cord after peripheral nerve injury. Scientific Reports. 2016; 6: 26427. 10.1038/srep26427 PMC4876408 27210057

[33] Vacca M, Celano G, Calabrese FM, Portincasa P, Gobbetti M, De Angelis M. The controversial role of human gut Lachnospiraceae. Microorganisms. 2020; 8: 573. 10.3390/microorganisms8040573 PMC7232163 32326636

[34] Hexun Z, Miyake T, Maekawa T, Mori H, Yasukawa D, Ohno M, *et al*. High abundance of Lachnospiraceae in the human gut microbiome is related to high immunoscores in advanced colorectal cancer. Cancer Immunology, Immunotherapy. 2023; 72: 315–326. 10.1007/s00262-022-03256-8 PMC10991469 35869338

[35] Kim K, Lee S, Park SC, Kim NE, Shin C, Lee SK, *et al*. Role of an unclassified Lachnospiraceae in the pathogenesis of type 2 diabetes: a longitudinal study of the urine microbiome and metabolites. Experimental & Molecular Medicine. 2022; 54: 1125–1132. 10.1038/s12276-022-00816-x PMC9440228 35931735

[36] Nie K, Ma K, Luo W, Shen Z, Yang Z, Xiao M, *et al*. Roseburia intestinalis: a beneficial gut organism from the discoveries in genus and species. Frontiers in Cellular and Infection Microbiology. 2021; 11: 757718. 10.3389/fcimb.2021.757718 PMC8647967 34881193

[37] Wu H, Huo Y, Fang W, Guo J, Wang X, Su L, *et al*. Gut microbiota and traumatic brain injury: insights from an antibiotic-free cohort. Frontiers in Microbiology. 2026; 16: 1697206. 10.3389/fmicb.2025.1697206 PMC12808345 41551652

[38] Sharma M, Vyas VK, Bhatt S, Ghate MD. Therapeutic potential of 4-substituted coumarins: a conspectus. European Journal of Medicinal Chemistry Reports. 2022; 6: 100086.

