# Supplementary material for: Gut microbial and metabolomic alterations associated with herpetic trigeminal neuralgia: an exploratory multi-omics study
Source: J Oral Facial Pain Headache. 2026 Sep 12;40(5):130–45. doi: 10.22514/jofph.2026.067 (PMC13678559; doi:10.22514/jofph.2026.067)
Supplement: Supplementary file 1 [file Supplementary-Figs.docx]

Supplementary material

**Supplementary Fig. 1. LASSO feature selection and ROC curve analysis for HTN diagnosis.** (a) Cross-validation curve for LASSO. (b) The ROC curve, which was used to evaluate the diagnostic performance of the LASSO screening model in distinguishing HTN patients from healthy controls in the test set. ROC: receiver operating characteristic; AUC: area under the curve.

**Supplementary Fig. 2.** **WGCNA analysis of HTN-related genes: soft-thresholding power selection and correlation between GS and MM in the blue and brown modules.** (a) Scatter plot of GS versus MM for the blue module in the WGCNA analysis. (b) Scatter plot of GS versus MM for the brown module in the WGCNA analysis. (c) The soft threshold selection plot for WGCNA analysis. Scale independence and Mean connectivity. WGCNA: weighted gene co-expression network analysis; HTN: Herpetic trigeminal neuralgia; GS: gene significance; MM: module membership.
